# Supplementary material for: Corneal Confocal Microscopy Predicts Cardiovascular and Cerebrovascular Events and Demonstrates Greater Peripheral Neuropathy in Patients with Type 1 Diabetes and Foot Ulcers
Source: Diagnostics (Basel). 2023 Aug 29;13(17):2793. doi: 10.3390/diagnostics13172793 (PMC10486928; doi:10.3390/diagnostics13172793)
Supplement: Supplementary file 1 [file diagnostics-13-02793-s001.zip › diagnostics-2516144-supplementary.pdf]

Supplementary Figure S1. ROC curves for CNFD, CNBD, CNFL, VPT, CT and WT for T1D-DPN.

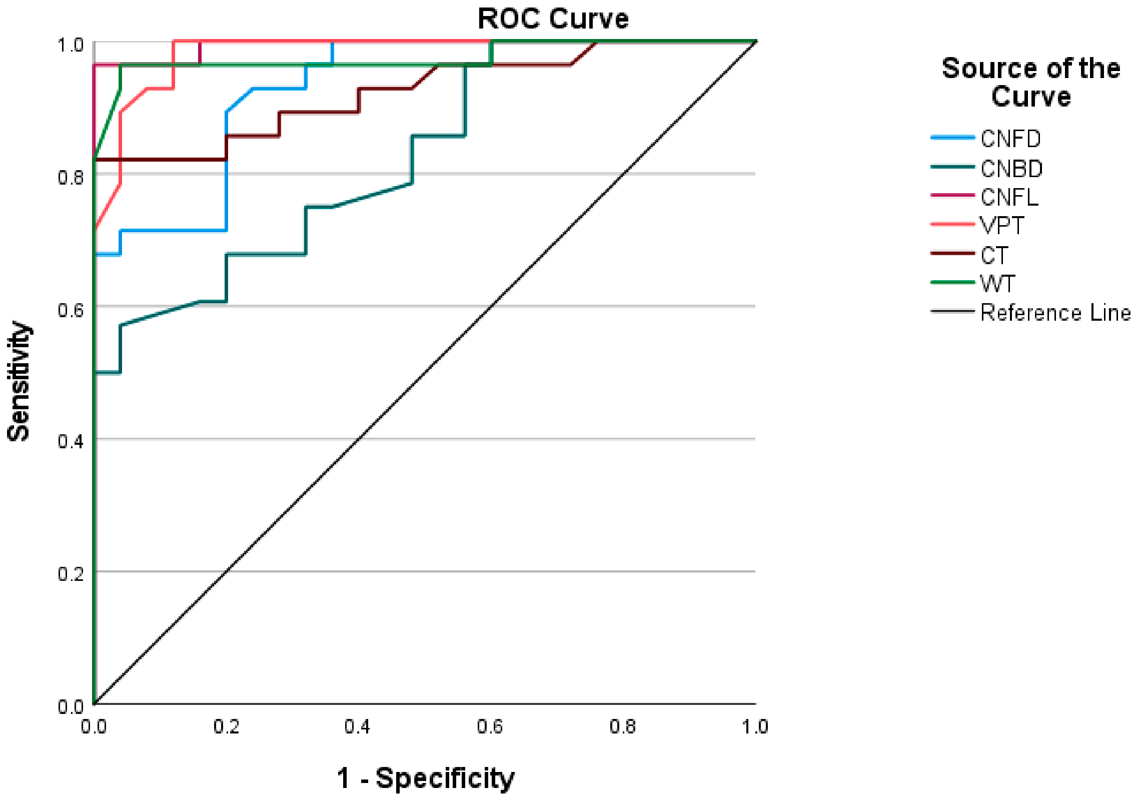

ROC – Receiver operating characteristic; CNFD – Corneal nerve fibre density; CNBD – Corneal nerve branch density; CNFL – Corneal nerve fibre length; VPT – Vibration perception threshold; CT – Cold Threshold; WT – Warm Threshold; T1D-DPN – type 1 diabetes with diabetic peripheral neuropathy.

Supplementary Table S1. ROC analysis with AUC, optimal cut off, sensitivity and specificity with 95% confidence interval in T1D with and without DPN.

	**Optimal Cut-off**	**Sensitivity**	**Specificity**	**AUC (95% CI)**	***p* Value**
CNFD (no./mm^2)	18.3	0.86	0.80	0.93 (0.87-0.99)	<0.001
CNBD (no./mm^2)	17.5	0.75	0.68	0.83 (0.72-0.93)	<0.001
CNFL (mm/mm^2)	16.2	0.96	0.92	0.98 (0.96-1.00)	<0.001
VPT (Volts)	12.1	0.93	0.88	0.98 (0.95-1.00)	<0.001
CT (°C)	23.5	0.82	0.92	0.92 (0.85-0.99)	<0.001
WT (°C)	39.9	0.82	0.88	0.92 (0.85-0.99)	<0.001

ROC – Receiver operating characteristic; AUC – area under the curve; CNFD – Corneal nerve fibre density; CNBD – Corneal nerve branch density; CNFL – Corneal nerve fibre length; VPT – Vibration perception threshold; CT – Cold Threshold; WT – Warm Threshold; T1D – Type 1 diabetes; T1D-DPN – Type 1 diabetes with diabetic peripheral neuropathy.

Supplementary Table S2a. Associations with incident cardiovascular events over 3 years in patients with T1D based on individual parameters with Cox regression analysis.

**Parameters**	**χ^2 Value**	**HR (95% CI)**	***p* Value**
Age$^{\alpha}$	5.6	1.06 (1.01-1.12)	0.025
Gender (male)	6.1	4.03 (1.15-14.08)	0.030
Duration of T1D$^{\alpha}$	8.9	1.07 (1.02-1.13)	0.004
CNFD$^{\beta}$	17.1	1.29 (1.10-1.50)	0.001
CNBD$^{\beta}$	15.1	1.22 (1.06-1.40)	0.005
CNFL$^{\beta}$	16.4	1.32 (1.09-1.60)	0.005
SNCV$^{\beta}$	9.6	1.11 (1.02-1.21)	0.013
SNAP$^{\beta}$	8.5	1.36 (1.03-1.81)	0.031
WT$^{\alpha}$	7.2	1.16 (1.04-1.29)	0.008
CT$^{\beta}$	13.5	1.20 (1.06-1.36)	0.004
VPT$^{\alpha}$	9.7	1.11 (1.04-1.18)	0.002

χ^2 – Chi-square value; HR – hazard ratio; 95% CI- 95% confidence interval; CNFD – Corneal nerve fibre density; CNBD – Corneal nerve branch density; CNFL – Corneal nerve fibre length; CT – Cold Threshold; WT – Warm Threshold; SNCV – Sural Nerve Conduction Velocity; SNAP – Sural Nerve Amplitude; T1D – Type 1 diabetes; VPT – Vibration perception threshold

α - Increased / higher value was associated with increased incidence of cardiovascular and cerebrovascular events
β - Decreased / lower value was associated with increased incidence of cardiovascular and cerebrovascular events

Supplementary Table S2b. Associations with incident cerebrovascular events over 3 years in patients with T1D based on individual parameters with Cox regression analysis.

**Parameters**	**χ^2 Value**	**HR (95% CI)**	***p* Value**
Age[α]	3.4	1.05 (0.99-1.11)	0.074
Gender (male)	8.1	5.99 (1.34-26.32)	0.019
Duration T1D[α]	4.3	1.05 (1.00-1.10)	0.043
CNFD[β]	16.8	1.29 (1.10-1.50)	0.001
CNBD[β]	16.6	1.25 (1.07-1.46)	0.004
CNFL[β]	18.1	1.38 (1.10-1.72)	0.005
SNCV[β]	9.9	1.12 (1.03-1.23)	0.012
SNAP[β]	10.1	1.46 (1.04-2.04)	0.026
WT[α]	6.5	1.15 (1.03-1.28)	0.011
CT[β]	10.4	1.17 (1.04-1.31)	0.008
VPT[α]	9.6	1.11 (1.04-1.19)	0.002

χ^2 – Chi-square value; HR – hazard ratio; 95% CI- 95% confidence interval; CNFD – Corneal nerve fibre density; CNBD – Corneal nerve branch density; CNFL – Corneal nerve fibre length; CT – Cold Threshold; WT – Warm Threshold; SNCV – Sural Nerve Conduction Velocity; SNAP – Sural Nerve Amplitude; T1D – Type 1 diabetes; VPT – Vibration perception threshold

α - Increased / higher value was associated with increased incidence of cardiovascular and cerebrovascular events
β - Decreased / lower value was associated with increased incidence of cardiovascular and cerebrovascular events
